# The future of departments of animal sciences in Argentina

**DOI:** 10.1093/af/vfaa021

**Published:** 2020-07-23

**Authors:** Gustavo Jaurena, María B Boveri

**Affiliations:** Universidad de Buenos Aires, Facultad de Agronomía, Departamento de Producción Animal, Buenos Aires, Argentina

**Keywords:** animal science, Argentina, beef, dairy, education

**Implications**

- Cattle production systems are facing dramatic changes due to increased social demands for cheap, safe, and high-quality products with less environmental impacts.
- Social demands are boosted by a not well-educated perception of agricultural productive systems and legitimate concerns about environmental and animal welfare issues.
- Future professionals in animal sciences will need traditional and new skills, such as environmental impact assessment, systems analysis theory, modeling, animal welfare, and bioethics.
- The university system in Argentina will have to respond with new and innovative research capabilities.

## Introduction

**Figure 1.**
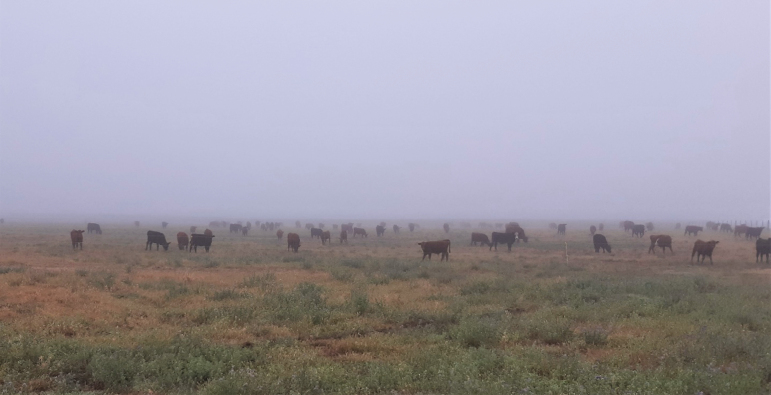
Beef cattle grazing in the province of Buenos Aires (Argentina).

Argentina is recognized for its high-quality beef, as well as its important dairy production sector. Many people probably think of beef production in Argentina as endless plains of grasslands and pastures where large herds of cattle graze peacefully at their own pace all year long (Figure 1). However, in the real world, beef and dairy production systems and, more generally, the current animal production systems are the result of complex interactions among the availability of natural resources and the environmental impact of cattle, cultural traditions, technical expertise, technological availability, social demands, and constraints, as well as economic pressures.

Universities in Argentina are the outcome of their European background and their later evolution due to global changes, local needs, and restraints. The aim of this article is to bring about a brief description of Animal Science Departments in Argentina´s Universities, as well as the authors’ thoughts on future teaching and researching challenges in these universities, facing changing social demands, and needs in relation to animal production.

## Relevant Features of Argentina

Argentina is placed in the extreme south of South America with an effective sovereign territory of 2,780,400 km^2^ (Instituto Geográfico Nacional, 2019), with ca. 45,000,000 inhabitants (2019). It is organized under a republican, democratic, representative, and federal government.

Education in Argentina involves four levels, the first three compulsory: initial (kindergarten from 4 or 5 years of age) and 12 years of primary and secondary school. Education is free for all levels, as well as for tertiary instruction under the public system, and private education institutions under governmental supervision are also available. The illiteracy rate is ca. 2% and, according to UNESCO, education in Argentina guarantees equality and favors multiethnic population education and special education for people with impaired abilities. Despite this, ca. 55% of students leave the secondary education system, which results in 50% of persons older than 25 years without having finished this level (Ministerio de Educación y Deportes, 2017).

In Argentina, there is a high demand for higher education studies (ca. 6,000 university students for every 10^6^ habitants in part because public universities are free of charge (even the very good ones) and because there are no admission examinations. Only about 24% of the adult populations have a complete superior education (Fanelli and Adrogué, 2018). The system would be expected to have positive effects in terms of equity facilitating the access for students from low income families, but, on average, only 20% and 50% of youngsters from low and medium socioeconomical levels, respectively have access to university studies. The rate climbs to 80% for the high-class levels. Besides, only ca. 11% of students complete their university studies within an average duration of 7–8 years (Nino, 2012). The graduation rate is higher for women and is positively associated with family income. Among the main reasons that account for the high abandonment rate are family income and the fact of being the first generation attending to university studies (Fanelli and Adrogué, 2018).

Employment statistics show that graduates from higher education have lower unemployment rates and lower risk to fall in the informal economy. Besides, urban employees with higher education receive higher payment than men or women with secondary instruction (García De Fanelli, 2016). Unfortunately, there are no available studies comparing different professions or types of degrees.

## The Argentinean University System

**Figure 2.**
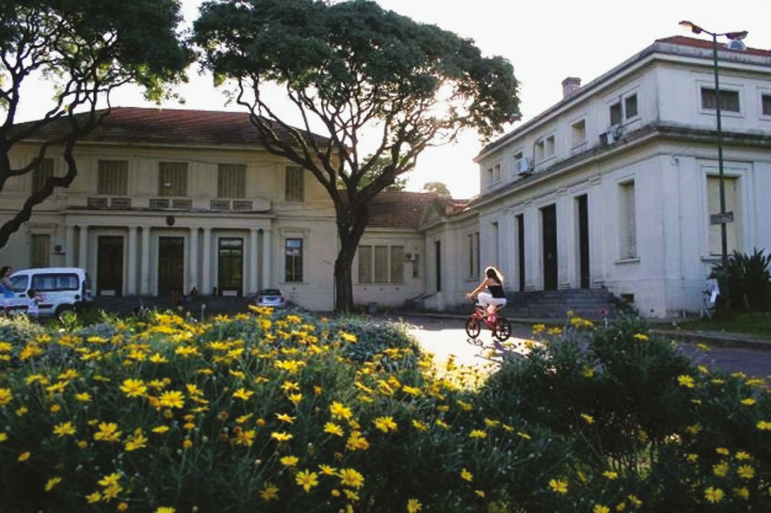
School of Agriculture—University of Buenos Aires.

Argentina has a mature and consolidated higher education system that offers a high-quality university with several reputable public and private institutions under the Academic Ranking of World Universities (ARWU rank 2019; e.g., Universidad de Buenos Aires, 201–300, Figure 2; Universidad de la Plata, 701–800; Universidad de Córdoba, 801–900) or under the global QS ranking or by different scores that consider the opinions of graduates and employers, as well as the academic reputation and employability of graduates.

In Argentina, the university system must fulfill functions in education, science, and technology and in social extension (Higher Education Law 24.521/95; Ley de Educación Superior, 1995). The public system covers the totality of the territory with at least one public university in each province. The public university governance includes a collegiate body, with representatives of professors, graduates, and students (Superior Council), that is in charge of appointing a Rector. Likewise, every school is under a similar organizational structure (dean and board of directors). On the other hand, private universities used to have a vertical structure with administrative bodies in charge of their direction and academic staff dealing with educational issues (García De Fanelli, 2016). The most widespread organizational structure is based on a chair system grouped around a disciplinary topic such as animal nutrition. Each chair position is covered by a professor who is accompanied by a team of professionals with appointments in research, extension, and lecturing in one or several courses.

By 2017, the whole system involved 2,005,152 undergraduate and graduate students (public: 1,584,392 and private: 420,760 students; 18.2% in careers of the applied science area) and 159,345 postgraduate students (public: 122,829 and private: 36,516 students; Ministerio de Educación, Cultura, Ciencia y Tecnología, 2018). These students were allocated among 131 institutions (public national: 61; public provincial: 5; private 63; international: 2) that employed 189,218 professors and readers (full time = 11%; between 10 and 20 h per week = 23%; up to 10 h per week = 66%) and 53,674 administrative and technical supportive staff.

**Figure 3.**
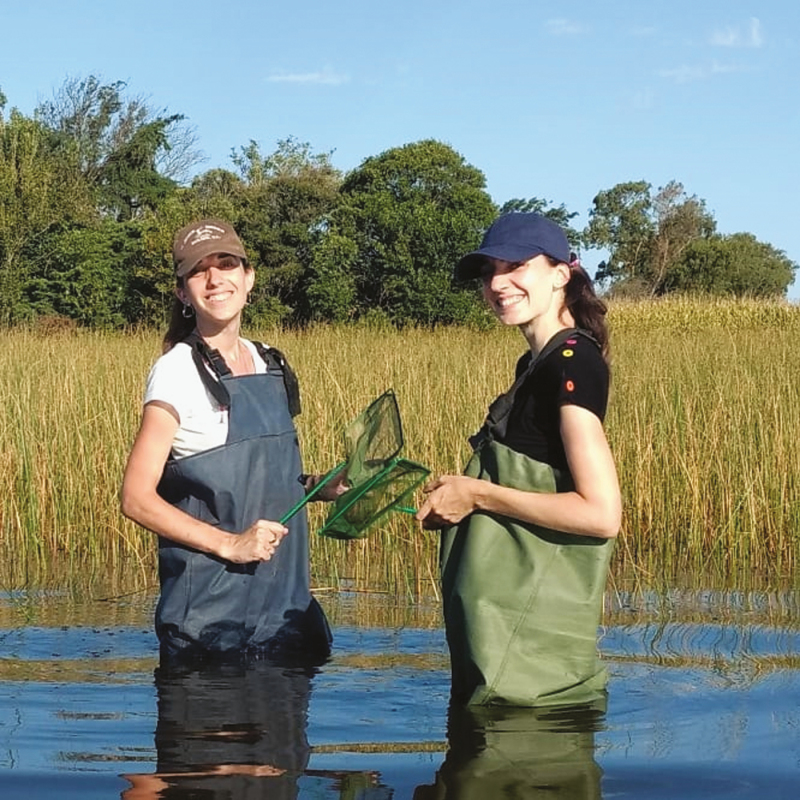
Photo of students at the School of Agriculture (University of Buenos Aires).

Student distribution is highly concentrated in the central part of Argentina with 24% of the total number of students attending an institution within the city of Buenos Aires; 24% in the province of Buenos Aries, and 13% in the province of Cordoba. In addition, the University of Buenos Aires accounts for 15% of the total number of students (https://informacionestadistica.rec.uba.ar/graficos_facultades.html), representing 58% of the total within the city of Buenos Aires (Figure 3).

**Figure 4.**
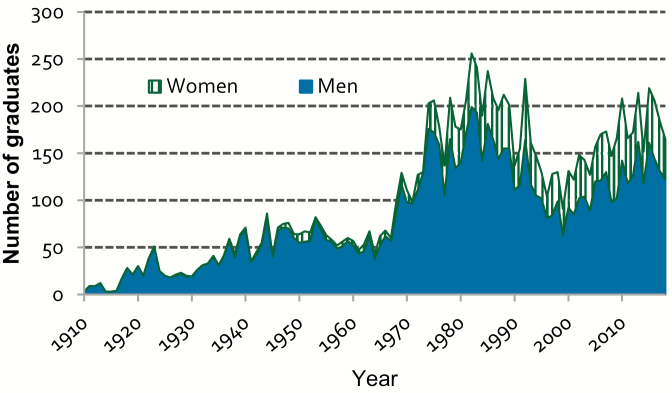
Number of men and women graduated from the School of Agriculture of the University of Buenos Aires (1908–2018).

Women participation in agriculture careers has been increasing steeply during the past century, reaching 30% of graduates in 2017 for the whole country (http://estadisticasuniversitarias.me.gov.ar/#/seccion/1). At the School of Agriculture (University of Buenos Aires), women graduation rate exceeded 20% since 1980 and has stabilized ca. 30% since year 2000 (Figure 4). Nowadays, 57% of the teaching and researchers positions are held by women, and noticeable discrepancies between genders in relation to project directions or areas of research do not exist (Kantolic and Gally, 2018).

At the present time, there are 66 universities offering degrees related with agriculture or animal science: 5/6 year degree in agricultural sciences, “Ingeniero Agrónomo” (28,348 students); animal production specialist (850 students); veterinarians (25.568 students; http://estadisticasuniversitarias.me.gov.ar/#/seccion/1).

**Figure 5.**
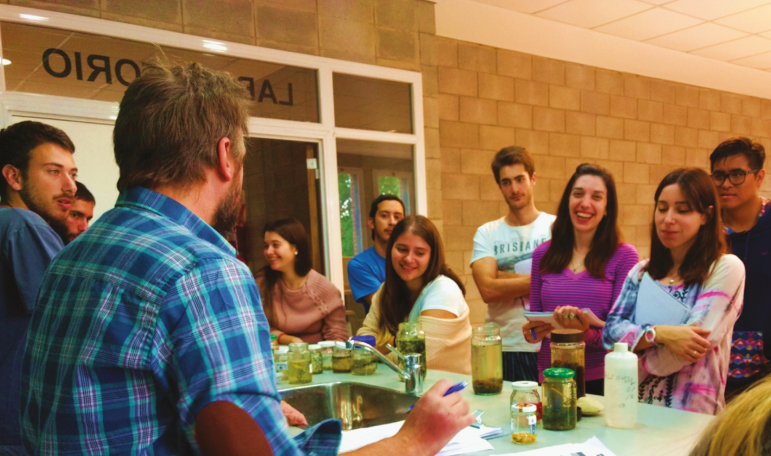
Students attending a class in the School of Agriculture (University of Buenos Aires).

The animal science curricula in agriculture-related careers usually includes core basic courses in animal anatomy and physiology, forage production, utilization and conservation, animal nutrition and feeding, animal breeding, and reproduction. After these basic courses, students follow courses in dairy and beef science, poultry and swine production, and other courses of regional or particular interest (Figure 5).

Aside from the university system, the technological and scientific system linked with agricultural issues is completed by the action of the National Institute of Agricultural Technology (INTA, “Instituto Nacional de Tecnología Agropecuaria”), and CONICET (National Scientific and Technical Research Council). In addition to the above-mentioned institutions, provincial institutes of scientific and technology development exist.

The INTA is a decentralized public body under the orbit of the Argentine Ministry of Agriculture, Livestock and Fisheries of the Nation with operational and financial autarchy, whose institutional goals are: “....to promote and invigorate agricultural research and extension to accelerate the technification and improvement of the agrarian enterprise and rural life.” It has a presence countrywide through 15 regional centers, 52 experimental stations, more than 350 extension units, and research centers (6) and institutes (22). It employs nearly 7,000 staff, of which 2,000 are R&D professionals, 2,000 are extension specialists, 2,400 are technical and support personnel, and 600 are scholarship researchers. Approximately, 700 are staff doing research in animal science disciplines (C. Roig, personal communication). CONICET is the main science and technology fostering agency with operations across the whole country with more than 10,000 researchers, 11,000 doctoral and postdoctoral fellows, 2,600 technicians and professional support staff, and 1,500 administrative employees.

The Argentine research and development system employs 109,460 people (189 researchers for every 10^5^ habitants), of which 76% (83,190 including research scholarship holders) are researchers (43% with a postgraduate degree). CONICET employs 21,640 persons (17,284 develop their research in public universities and public universities employ 44,153 scientists, of which 56–60% are women; Quattrone et al., 2017).

Argentine expenditures in the entire education system is ca. 6.1% of gross domestic product (GDP; in 2015; Ministerio de Educación y Deportes, 2017, p. 71) and in higher education was 0.8% of GDP in 2017 (Cetrángolo and Curcio, 2017). In addition, investment in research and development for 2017 was ca. 0.55% of GDP; financial source: 73% public sector, 18% private, and 9% external, of which 48% was executed by public organizations, 25% by public universities, and 25% by companies (Quattrone et al., 2017, p. 4).

Regarding scientific productivity, between years 2000 and 2019, a total of 108 articles appeared in the Scopus database (survey in discipline Agricultural and Biological Sciences), and 282 peer-reviewed, full articles appeared in the *Argentine Journal of Animal Science* (“Rev. Argentina de Prod. Animal, RAPA”, online ISSN 2314-324X; “Asoc. Argentina de Prod. Animal; AAPA”). Furthermore, a total of 6,195 extended summaries (ca. 1,000 words) were sent to be published in the proceedings of the AAPA annual meeting (Table 1). The RAPA is a peer-reviewed journal (not indexed) that gathers good quality articles of relevance for Argentina and Latin America. The large discrepancy between the number of published articles (108 + 282) and extended summaries presented in the annual meetings of AAPA is remarkable, indicating a gap in producing high-quality, full-text publications among the participants. Behind this difference, it may be speculated that a lack of adequate stimulus for many researchers to fully complete the scientific process exists because, in many institutions, researchers are not compelled to publish as an indicator of work productivity or multiple simultaneous demands compete for its time (e.g., teaching, administrative, and extension).

**Table 1.** Number of scientific publications from 2000 to 2019 within Agricultural and Biological Sciences database in Scopus and within the Argentine Association of Animal Production publications database classified by discipline

		AAPA journal	
	Scopus^*a*^	Full articles	Proceedings
Total number of publications	108	282	6,195
Relative contributions (%)			
Forages and pastures	28	28	32
Animal production systems^*b*^	24	8	14
Health in animal production	13	6	6
Nutrition and feeding	6	19	23
Breeding and reproduction in animal production	5	16	10
Technology in animal products	0	12	9
Others subtotal	24	12	5
Environmental impact^*c*^	21	0	0
Animal welfare^*c*^	3	0	0

^*a*^Using Scopus Agricultural and Biological Sciences as discipline, Argentina as the country of affiliation, and animal science or animal production as key words.

^*b*^Previously included environmental issues in animal production.

^*c*^Recently created sections.

## Present and Future Challenges

In Argentina, demands on animal production systems and agriculture, in general, resemble what has been happening throughout the world—there is increased pressure on animal production systems to use fewer natural resources and generate higher-quality products with fewer environmental impacts to meet changing consumer demands. These production, environmental, and societal issues are gaining publicity and persistency in the news media on political agendas and across the education system in Argentina.

Consequently, we think that the analysis of animal production topics should be moving away from a multifactor-single-objective conventional view to a more comprehensive multifactor-multiobjective viewpoint with simultaneous consideration of factors such as soils or climate and multiobjectives as quality and quantity of milk production or environmental impacts. This course of action will demand a more interdisciplinary, transdisciplinary, and multidisciplinary approach in research and teaching. Hereafter, the present complexity of cattle production system studies and interactions with other social and biophysical agents will demand formal system analysis theory and modeling instruction and research. These elements should orient specific disciplinary activities and provide future graduates with effective knowledge, skills, and abilities to enhance their decision-making processes and application of their new knowledge from a broader range of disciplines. This interdisciplinary approach is almost absent in the current curricula and research agenda within animal science training programs in Argentina (Jaurena et al., 2013; Ginies and Jaurena, 2015).

While the standard animal science curricula will remain essentially the same, future animal science professionals must acquire competences in animal welfare, environmental impact assessment, social perception, and food safety. Above all, new biotechnologies (e.g., advanced genetics or genomics technologies to identify genetically superior sires at an earlier age), reproduction technologies (e.g., artificial insemination, embryo transfer, cloning) or nutrition advances (e.g., growth promotants, alternative feedstuffs) provide alternatives to conventional animal production systems. These alternatives will demand special attention not only due to the technical training requirements, but also due to their bioethical consequences. The novel technologies for animal production have captured considerable public attention and resulted in significant amounts of human and economic resource investments.

Because many of these issues are beyond the scope of traditional animal science experts and present departments, the training program for future animal scientists will need to include experts from other disciplines and departments to provide future animal scientists and practitioners with a broader and more appropriate educational basis.

The current availability and access to the internet, as well as the increased familiarity of students with web information, will facilitate the delivery of a wider range of contents through e-learning devices and virtual reality. In this respect, the COVID-19 pandemic has accelerated and forced the national university system to cope with the delivery of online courses and teaching activities. Within this context, the present challenge is not how to get information but how to differentiate accurate and science-based information from fiction or inaccurate information. The problem of free information on the internet is a particularly sensitive issue in social media environments where the news media, social influencers, and even educators or some societal leaders do not use objective or science-based information.

Up until now, the scientific and technical research in Argentina has been supported mainly by public funds, but joint public–private initiatives are fruitful associations not only for the genuine source of funds for research but also because it fosters mutually beneficial feedback (e.g., specific studies and customer-tailored courses).

There has been a historical lack of sufficient investment in facilities and equipment to meet the specific demands of animal science research and training of graduate and postgraduate students. The lack of up-to-date infrastructure and laboratory equipment, along with the lack of highly capable technical personnel not only constrains the development of basic or applied research but also limits a more fruitful interaction of the university system with the private sector. This problem is not exclusively due to the relative lack of funding but also due to increased bureaucracy and burdensome policies.

Animal production is changing worldwide, as well as its relationship with consumers and especially with the public perception of food, animals, and the environment. Argentina is a salient player in agriculture and specifically in animal production with an important share of Argentina’s economy depending on agriculture and the production of animal-sourced foods. Future animal scientists and practitioners will be required to fulfill traditional technical capacities alongside novel competences to deal with complex productive systems within highly demanding social environments. Hence, higher education institutions will have to face the challenge of adapting their institutional structures, educational curricula, outreach activities, and research topics to facilitate the acquisition and maintenance of these competences.

## About the Authors

Gustavo Jaurena is associate professor of animal nutrition in the Department of Animal Sciences and co-director of the Animal Science Program at the School of Agriculture of the University of Buenos Aires. He is Engineer in Agriculture (animal science major; University of Buenos Aires), and he earned a master’s degree in animal science at the Pontifical Catholic University of Chile and the PhD degree at the University of Wales. He was President of the Argentine Association of Animal Production from 2013 to 2015. His current research is focused on nutritional strategies to mitigate greenhouse gas emissions from ruminants. He also assesses animal feedstuffs by coordinating the “Program for the Improvement of Forage and Feedstuffs Quality Assessment,” an interinstitutional initiative that contributes to the improvement of analytical work of animal nutrition laboratories.

María Boveri is professor in charge of the Aquaculture and Aquatic Ecology group and director of the Department of Animal Science in the School of Agriculture at the University of Buenos Aires. She has a minor in geography and a degree in agriculture engineering. She earned a doctoral degree in agricultural science at the University of Buenos Aires and has developed professional activities in the private and academic sectors teaching graduate and postgraduate courses. Her research focuses on the environmental impact of anthropic activities on freshwater surface bodies and small wetland ecological services onto crops and cattle production systems.
